# Commentary: Trends and Development in Enteral Nutrition Application for Ventilator Associated Pneumonia: A Scientometric Research Study (1996–2018)

**DOI:** 10.3389/fphar.2019.01056

**Published:** 2019-09-19

**Authors:** Yuh-Shan Ho

**Affiliations:** Trend Research Centre, Asia University, Taichung, Taiwan

**Keywords:** Web of Science (WOS), Science Citation Index, trend research, SSCI, scientometric analyses

Chen et al. (2019) recently published a paper in the journal, entitled “Trends and development in enteral nutrition application for ventilator associated pneumonia: a scientometric research study (1996–2018).” Many of the related results presented in the original paper (Chen et al., 2019) are not acceptable because of the use of inappropriate method. Chen et al. stated in dataset that “Regarding data collection, the following retrieval strategy was developed: ‘TS = ([ventilator-associated pneumonia or ventilator-associated pneumonia or ventilator-acquired pneumonia or ventilator-acquired pneumonia] and [enteral nutrition]).’ Language was set as ‘English’, literature category as ‘Article’, and time from 1996/01/01 to 2018/8/23.”

Results in the original paper (Chen et al., 2019) cannot be repeated by using the same methods. In total, 126 articles were found.

Web of Science (WoS) includes

1. Web of Science Core Collection
2. Data Citation Index
3. Current Contents Connect
4. BIOSIS^®^ Citation Index
5. Biological Abstracts^®^
6. CABI
7. Chinese Science Citation Database
8. FSTA
9. Inspec^®^
10. MEDLINE^®^
11. SciELO Citation Index
12. Zoological Record^®^
13. Derwent Innovations Index
14. Journal Citation Reports
15. Essential Science Indicators

Web of Science Core Collection includes

1. Science Citation Index Expanded (SCI-EXPANDED)
2. Social Sciences Citation Index (SSCI)
3. Arts and Humanities Citation Index (A&HCI)
4. Conference Proceedings Citation Index—Science (CPCI-S)
5. Conference Proceedings Citation Index—Social Science & Humanities (CPCI-SSH)
6. Book Citation Index—Science (BKCI-S)
7. Book Citation Index—Social Sciences & Humanities (BKCI-SSH)
8. Emerging Sources Citation Index (ESCI)

Web of Science Core Collection: Chemical Indexes include:

1. Current Chemical Reactions (CCR-EXPANDED)
2. Index Chemicus (IC)

Since there are many levels of databases as mentioned above, the authors should choose the appropriate databases for their research. For instance, Emerging Sources Citation Index (ESCI) complements the highly selective indexes by providing earlier visibility for sources under evaluation as part of SCIE, SSCI, and A&HCI’s rigorous journal selection process (http://wokinfo.com/products_tools/multidisciplinary/esci/). Web of Science Core Collection: chemical indexes as well as SSCI, A&HCI, ESCI, CPCI-S CPCI-SSH, BKCI-S, and BKCI-SSH are inappropriate for “Trends and development in enteral nutrition application for ventilator associated pneumonia: a scientometric research study (1996–2018)” (Chen et al., 2019).

One appropriate method is to use SCI-EXPANDED with searching keywords (“ventilator-associated pneumonia” or “ventilator-acquired pneumonia”) and (“enteral nutrition”) from 1996 to 2018. Language of “English” and document type of “article” were considered. This method resulted in 109 articles in SCI-EXPANDED. The SCI-EXPANDED is designed for researchers to find published literatures, not used for bibliometric studies (Ho, 2018a; Ho, 2018b; Ho, 2019).

Thus, it is always necessary use an accurate bibliometric treatment when using the Web of Science database (Ho, 2018a; Ho, 2018b; Ho, 2019). It was pointed out that the documents, which can only be searched out by *KeyWords Plus*, were irrelevant to “enteral nutrition application for ventilator associated pneumonia” (Fu and Ho, 2015). Ho’s group was the first to propose “front page” as a filter to improve the bibliometric method (Fu et al., 2012; Fu and Ho, 2014; Ho and Fu, 2016). Only documents with searching keywords in their “front page,” including the article title, the abstract, and the author keywords were considered.

As a result, 56 articles (51% of the 109 articles) had search keywords in their “front page” while 35 articles (32%) and 24 articles (22%) did not include (“ventilator-associated pneumonia” or “ventilator-acquired pneumonia”) and “enteral nutrition” in their “front page,” respectively. For example, highly cited review with 100 or more total citations from Web of Science Core Collection since publication to the end of 2017 (TC_2017_ ≥ 100) (Hsu and Ho, 2014), “Incidence of and risk factors for ventilator-associated pneumonia in critically ill patients” (Cook et al., 1998), did not have “enteral nutrition”; “Tracheobronchial aspiration of gastric contents in critically ill tube-fed patients: frequency, outcomes, and risk factors” (Metheny et al., 2006) did not have “ventilator-associated pneumonia” and “ventilator-acquired pneumonia.” “ISBI practice guidelines for burn care” (Ahuja et al., 2016) did not have any searching keywords in its “front page.” By using the “front page” as a filter, it will avoid introducing unrelated articles for analysis (Fu et al., 2012; Ho, 2018c). Similar rebuttals have also been published in Environmental Science and Pollution Research (Ho, 2018a), Renewable and Sustainable Energy Reviews (Ho, 2018c), and Journal of Soils and Sediments (Ho, 2019) in previous years.

Chen et al. (2019) published the bibliometric paper in European Planning Studies using an inappropriate method; this may result in misleading the journal readers (Ho, 2018b; Ho, 2019). In my opinion, Chen et al. could have provided a greater accuracy and information about their data if they understood Web of Science beforehand. In addition, Chen et al. used only 124 papers published from 1996 to 2018 for their study. It is inappropriate to use such limited numbers of articles for bibliometric studies from a statistics point of view.

## Author Contributions

Y-SH confirms being the sole contributor of this manuscript.

## Conflict of Interest Statement

The author declares that the research was conducted in the absence of any commercial or financial relationships that could be construed as a potential conflict of interest.
